# Heme Oxygenase-1 Is Upregulated during Differentiation of Keratinocytes but Its Expression Is Dispensable for Cornification of Murine Epidermis

**DOI:** 10.3390/jdb11010012

**Published:** 2023-03-10

**Authors:** Marta Surbek, Supawadee Sukseree, Attila Placido Sachslehner, Dragan Copic, Bahar Golabi, Ionela Mariana Nagelreiter, Erwin Tschachler, Leopold Eckhart

**Affiliations:** 1 Department of Dermatology, Medical University of Vienna, 1090 Vienna, Austria; 2 Clinical Division of Nephrology and Dialysis, Department of Internal Medicine III, Medical University of Vienna, 1090 Vienna, Austria

**Keywords:** keratinocytes, epidermis, differentiation, heme, heme oxygenase, hemoprotein, transglutaminase, loicrin, oxidative stress, iron

## Abstract

The epidermal barrier of mammals is initially formed during embryonic development and continuously regenerated by the differentiation and cornification of keratinocytes in postnatal life. Cornification is associated with the breakdown of organelles and other cell components by mechanisms which are only incompletely understood. Here, we investigated whether heme oxygenase 1 (HO-1), which converts heme into biliverdin, ferrous iron and carbon monoxide, is required for normal cornification of epidermal keratinocytes. We show that HO-1 is transcriptionally upregulated during the terminal differentiation of human keratinocytes in vitro and in vivo. Immunohistochemistry demonstrated expression of HO-1 in the granular layer of the epidermis where keratinocytes undergo cornification. Next, we deleted the *Hmox1* gene, which encodes HO-1, by crossing *Hmox1*-floxed and *K14-Cre* mice. The epidermis and isolated keratinocytes of the resulting *Hmox1^f/f^ K14-Cre* mice lacked HO-1 expression. The genetic inactivation of HO-1 did not impair the expression of keratinocyte differentiation markers, loricrin and filaggrin. Likewise, the transglutaminase activity and formation of the stratum corneum were not altered in *Hmox1^f/f^ K14-Cre* mice, suggesting that HO-1 is dispensable for epidermal cornification. The genetically modified mice generated in this study may be useful for future investigations of the potential roles of epidermal HO-1 in iron metabolism and responses to oxidative stress.

## 1. Introduction

The epidermis of mammals has several essential functions, such as the reduction in water loss from the body, suppression of entry of chemical substances, resistance against mechanical stress and defense against pathogens [1,2,3,4,5]. The epidermal barrier against the environment is established by epithelial cells known as keratinocytes, which constitute more than 90% of cells in the epidermis [5,6].

The epidermis forms during embryonic development and is continuously regenerated postnatally by the differentiation of keratinocyes from stem cells [5,7,8]. Development of the epidermis starts from a single layer of epithelial cells covering the body surface. This primordial layer is subsequently covered by the periderm, consisting of specialized embryonic epithelial cells. The periderm prevents aberrant fusions between adhesion-competent epithelia [9] and provides a transient barrier of the skin epithelium during embryonic development [10]. In the main model species of mammalian skin biology, the mouse, the periderm forms on embryonic day E9.5. Keratinocyte proliferation and delamination lead to the formation of additional, suprabasal layers of keratinocytes. Around embryonic day E17.5, the epidermis begins to undergo cornification, which involves the expression of classical markers of epidermal barrier, such as filaggrin, loricrin and caspase-14 [11]. Upon formation of a barrier-competent stratum corneum [12], the periderm degenerates and is shed from the skin surface. In late embryonic development, the epidermis enters a homeostatic equilibrium of cell proliferation in the basal layer and cell death by cornification in the outermost granular layer, being connected by cell differentiation in the intermediate layers of the epidermis.

**Figure 1:**
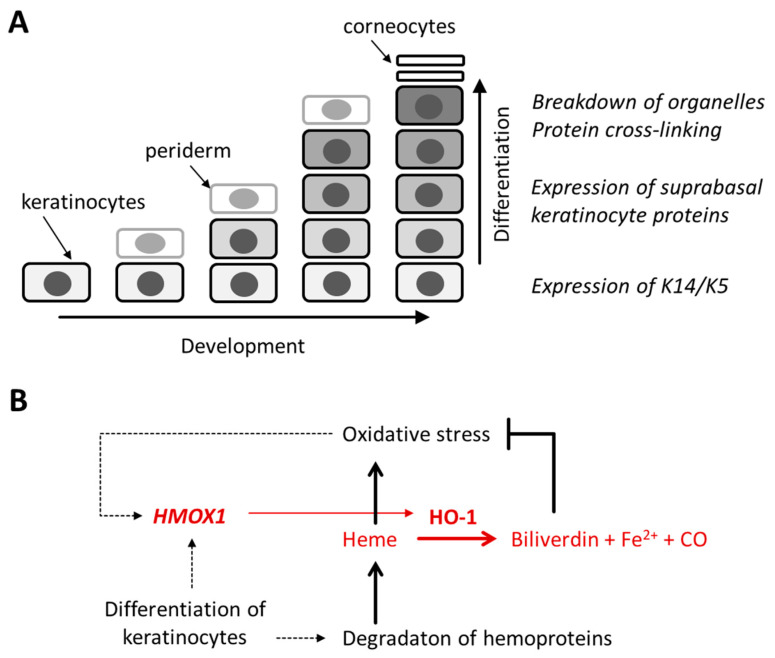
Hypothesis on the role of heme oxygenase-1 in the epidermis. (**A**) Schematic model of epidermal development and keratinocyte differentiation. K, keratin. (**B**) Regulation and function of heme oxygenase-1 (HO-1). The expression of the *HMOX1* gene is induced by oxidative stress in many cell types including keratinocytes. Differentiation of keratinocytes is hypothesized to alter the expression of *HMOX1* and to cause degradation of hemoproteins, thereby releasing heme. The latter is converted by HO-1 into biliverdin, ferrous iron (Fe^2+^) and carbon monoxide (CO).

Keratinocyte differentiation is a multi-step process which is tightly controlled by changes in gene expression and post-transcriptional events [7,8,13,14]. Differentiation involves the transcriptional upregulation of specific genes, accumulation and cross-linking of structural proteins and eventually the degradation of organelles [6,15]. Many of the keratinocyte differentiation-associated proteins, such as filaggrin and loricrin, have functions exclusively in the epidermis, whereas others are expressed either constitutively or in an inducible manner also in other tissues. Heme oxygenase-1 (HO-1), which is encoded by the orthologous genes *HMOX1* in humans and *Hmox1* in mice, is upregulated by exposure to oxidative stress in various tissues [16] but expressed also in apparently unstressed epidermis [17,18]. In the mouse, the promoter of the *Hmox1* gene is constitutively active in the suprabasal epithelial cells of the epidermis, tongue and esophagus [19]. HO-1 converts heme, a potent inducer of oxidative stress, into the anti-oxidant biliverdin, thereby contributing to the protection against damaging effects of oxidative stress. Additional products of HO-1-mediated breakdown of heme are ferrous iron and carbon monoxide [20] (Figure 1). The substrate of HO-1, heme, is an essential component of hemoproteins, such as cytochrome c and CYP450. Cytochrome c immunoreactivity decreases in the upper granular layer of the epidermis and disappears in the stratum corneum [21], likely reflecting the degradation of cytochrome c by one or more proteases active during cornification [6]. Assuming that the hydrolysis of hemoproteins releases free heme in terminally differentiated keratinocytes, we hypothesized that HO-1 might metabolize heme to allow for normal epidermal cornification.

To test the hypothesis that HO-1 is critically involved in terminal differentiation of epidermal keratinocytes, we determined the expression pattern of HO-1 in the skin of humans and mice, deleted *Hmox1* in keratinocytes of mouse skin and investigated the phenotype of mice lacking epithelial HO-1. 

## 2. Materials and Methods

### 2.1. Ethics Statement

Human tissue samples were obtained from abdominal skin reduction surgery procedures. Informed consent was obtained. The study was conducted according to the guidelines of the Declaration of Helsinki and approved by the Ethics Committee of the Medical University of Vienna (approval EK. 1969/2021, date of approval 18 November 2021). Mouse tissue samples were prepared from mice immediately after killing the animals. The Ethics Committee of the Medical University of Vienna decided that, in agreement with the national laws of Austria, a permission for killing mice for organ preparation was not required. 

### 2.2. Mice

*Hmox1^f/f^* mice [22] were crossed with *K14-Cre* mice [23] to generate *Hmox1^Δep^* (*Hmox1^f/f^ K14-Cre*) mice in which *Hmox1* is deleted in epidermal keratinocytes. The mice were kept on a 12-h light and dark cycle and had access to chow and water ad libitum. Genotyping of mice was performed using a PCR with the primers Hmox1-f, 5′-GGAGGTTGGAGCCAGGAATGATGAG-3′, and Hmox1-r, 5′-GAGTCTTCTTCCCCTTATGCACGTCA-3′, for *Hmox1* and Cre-f, 5′-TTTGCCTGCATTACCGGTCGATGCAAC-3′, and Cre-r, 5′-TGCCCCTGTTTCACTATCCAGGTTACGGA-3′ for the *Cre* transgene. For the preparation of tissue samples, mice were killed by cervical dislocation according to the animal welfare guidelines of the Medical University of Vienna, Austria. All animal procedures were conducted in agreement with the national laws. For gene expression analyses, the epidermis was separated from the dermis by dispase treatment and keratinocytes were cultured according to published protocols [24]. Briefly, after separation of the epidermis from tail skin by dispase treatment, the epidermal sheets of 4 mice per genotype were pooled and treated with trypsin. Isolated keratinocytes were suspended in keratinocyte growth medium 2 (KGM2, PromoCell, Heidelberg, Germany) and grown for 8 days before they were lysed with Trizol reagent.

### 2.3. Cell Culture

Human and mouse keratinocytes were isolated and cultured according to published protocols [24,25]. Human skin equivalents were generated as reported previously [26].

### 2.4. Histology, Immunohistochemistry and Immunofluorescence Analysis

Tissue samples were fixed in 7.5% formaldehyde (catalog number FN-1000-75-1, SAV Liquid Production GmbH, Flintsbach am Inn, Germany) overnight and embedded in paraffin. Hematoxylin and eosin (H&E) staining was done according to a standard protocol [27]. Immunohistochemistry and immunofluorescence analysis were performed according to published protocols [25,27] with antibodies against HO-1 (dilution 1:500, catalog number HPA000635, Sigma-Aldrich, St. Louis, MO, USA), loricrin (dilution 1:5000, Covance, Princeton, NJ, USA) and filaggrin (dilution 1:10,000, Covance, Princeton, NJ, USA), raised in rabbits. For immunohistochemistry, the sections were subsequently incubated with goat anti-rabbit immunoglobulin conjugated to horseradish peroxidase for 30 min. The color was developed with the Vectastain ABC-HRP kit, PK-4000 (Vector Laboratories, Burlingame, CA, USA). Nuclei were counterstained with hematoxylin. For immunofluoresence analysis, the sections were incubated with goat anti-rabbit immunoglobulin antibodies conjugated to Alexa-Fluor 546 (Molecular Probes, Eugene, OR, USA). Nuclei were labelled with Hoechst 33258 (Molecular Probes, Eugene, OR, USA). Rabbit immunoglobulin G (catalog number P120-101, Bethyl Laboratories, Montgomery, TX, USA) was used instead of the primary antibodies in the negative control experiments. Finally, the sections were mounted with Permafluor (Thermo Fisher, Waltham, MA, USA) and examined with an Olympus BX63 light microscope. Photographs were taken with an Olympus UC-90 camera.

### 2.5. Transglutaminase Activity Labeling on Tissue Sections

Tissue samples were embedded in optimal cutting temperature (OCT) compound (Scigen Scientific, Gardena, CA, USA) and snap-frozen in liquid nitrogen. Cryosections of 6 µm thickness were cut with a cryostat (Leica CM3050S) and stored at −80 °C. Transglutaminase activity was localized with an in situ assay according to a published protocol [28], with minor modifications. Briefly, cryosections were incubated with 5 µM Alexa-fluor-555-cadaverine (Thermo Fisher, Waltham, MA, USA), a substrate of transglutaminases, in 0.1 M Tris-HCl pH 7.4 in the presence of 5 mM CaCl_2_ for 2 h. In negative control experiments, CaCl_2_ was replaced by 5 mM EDTA. The reaction was stopped by incubating the slides in 25 mM EDTA in PBS for 5 min. Nuclei were labeled with 1 µg/mL Hoechst 33258 (Molecular Probes, Eugene, OR, USA). The sections were mounted with Permafluor (Thermo Fisher, Waltham, MA, USA) and examined with an Olympus BX63 microscope. Photographs were taken with an Olympus UC-90 camera.

### 2.6. Western Blot Analysis

Human skin equivalents were lysed in a buffer containing 50 mM Tris (pH 7.4), 2% SDS and complete protease inhibitor cocktail (Roche, Mannheim, Germany), and homogenized by sonication. After removal of insoluble debris by centrifugation, 25 μg protein was electrophoresed through a sodium dodecyl sulfate (SDS) polyacrylamide gel (Bio-Rad Laboratories, Hercules, CA, USA) and thereafter blotted onto a nitrocellulose membrane. Rabbit anti-HO-1 (dilution 1:500, catalog number HPA000635, Sigma-Aldrich, St. Louis, MO, USA) was used as the primary antibody and goat anti-rabbit immunoglobulin G conjugated to horseradish peroxidase (dilution 1:10,000, catalog number 1706515, Sigma-Aldrich, St. Louis, MO, USA) was used as the secondary antibody. Enhanced chemiluminescence reagent (catalog number 1859024, Thermo Fisher, Waltham, MA, USA) was used to develop the signal.

### 2.7. Quantitative RT-PCR Analysis

mRNA was isolated from tissues and cells and subjected to reverse-transcription quantitative PCR analysis according to published protocols [11,24]. The following primer pairs were used for PCRs of mouse *heme oxygenase 1* (*Hmox1*): 5′-GCCACCAAGGAGGTACACAT-3′ and 5′-GCTTGTTGCGCTCTATCTCC-3′, *glutamate-cysteine ligase catalytic subunit* (*Gclc*): 5′-GACTTCCTCATTCCGCTGTC-3′ and 5′-ATACCCCTTCCTTCCCATTG-3′, *filaggrin* (*Flg*): 5′-AAAAGATGTCCGCTCTCCTG-3′ and 5′-CTTCAGCGATGTCTTGGTCA-3′, *loricirin*: 5′-CCTGTGGGTTGTGGAAAGAC-3′ and 5′-TGGAACCACCTCCATAGGAA-3′ and *transglutaminase 1* (*Tgm1*): 5′-CTACTCTCGAGGCACCAACC-3′ and 5′-TGTGTCGTGTGCAGAGTTGA-3′. The expression levels of the target genes were normalized to the abundance of cDNA of the housekeeping gene *beta-2-microglobulin* (*B2m*), amplified with the primers 5′-ATTCACCCCCACTGAGACTG-3′ and 5′-TGCTATTTCTTTCTGCGTGC-3′, defining 1 arbitary unit as the mean of the expression in wildtype (*Hmox1^f/f^*) samples. Human cDNAs were amplified with primers specific for *HMOX1*: 5′-AAGATTGCCCAGAAAGCCCTGGAC-3′ and 5′-AACTGTCGCCACCAGAAAGCTGAG-3′, *FLG*: 5′-ATCTTCTCGGGAGCAGTCAA-3′ and 5′-CCTTTCAGTGCCCTCAGATT-3′, *LORICRIN*: 5′-GGAGTTGGAGGTGTTTTCCA-3′ and 5′-ACTGGGGTTGGGAGGTAGTT-3′ and *B2M*, 5′-GGGATCGAGACATGTAAGCAG-3′ and 5′-GAGCTACCTGTGGAGCAACC-3′. Data are presented as mean +/− standard deviation. The significance of difference between expression levels in *Hmox1^f/f^* and *Hmox1^Δep^* mouse tissue was determined with the unpaired two-sided t-test with *p* < 0.05 being considered significant. The significance of difference between the time points of human keratinocyte differentiation in vitro was determined with one way ANOVA analysis with *p* < 0.05 being considered significant.

### 2.8. Single-Cell RNA-Sequencing (scRNA-seq) Data Analysis

scRNA-seq data published previously [29] were analyzed for mRNAs *HMOX1*, *CYCS* and *LOR* (loricrin) in human epidermis. The skin samples were collected during abdominoplasty from 3 healthy donors and further processed using published protocols [29]. The data analysis was performed as described previously [29,30]. For data presentation, violin plots were generated, where the vertical axis represents log-transformed single-cell expression levels. The shape of each violin indicates the frequency of cells at different expression levels. Vertical lines represent maximum expression (log 2-fold change).

## 3. Results

### 3.1. Expression of HMOX1 Is Upregulated during Terminal Differentiation of Epidermal Keratinocytes 

**Figure 2:**
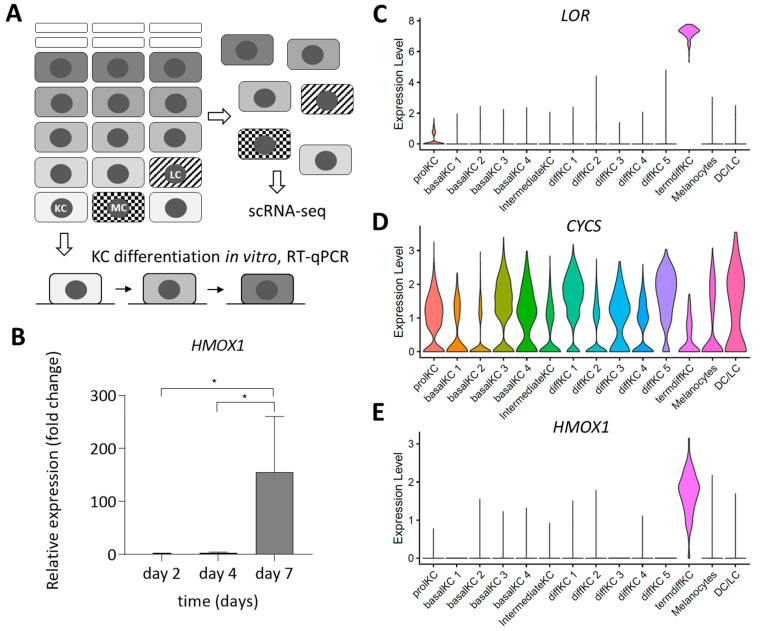
Heme oxygenase-1 (*HMOX1*) mRNA is upregulated during terminal differentiation of human epidermal keratinocytes. (**A**) Schematic depiction of experiments aimed at the determination of gene expression in differentiating keratinocytes. The epidermis consists largely of keratinocytes (KC) but also contains melanocytes (MC) and Langerhans cells (LC). KCs were isolated and induced to undergo differentiation in vitro by maintenance in confluent culture for up to 7 days, followed by reverse transcription-quantitative PCR (RT-qPCR) analysis. In separate experiments, cells were isolated and subjected to single-cell RNA sequencing (scRNA-seq). (**B**) RT-qPCR of *HMOX1* in human KCs differentiating in vitro. Relative expression levels are normalized to a reference gene. Asterisks indicate significant differences (*p* < 0.05), as determined by one way ANOVA. (**C**–**E**) Single-cell transcriptomic analysis of *Loricrin* (*LOR*) (**C**), *Cytochrome c* (*CYSC*) (**D**) and *HMOX1* (**E**) expression in cells of the human epidermis. A published dataset was analyzed, using the same designation of cell populations [30]. The violin plots show the log-transformed single-cell expression level on the vertical axis and the frequency of cells at different expression levels in the horizontal dimension. DC, dendritic cells; diffKC, differentiating KC; LC, Langerhans cells; prolKC, proliferating keratinocytes; termdiffKC, terminally differentiated keratinocytes.

The expression of *HMOX1* was investigated in human keratinocytes differentiating in vitro and in vivo (Figure 2A). First, primary epidermal keratinocytes were maintained in confluent culture for 7 days to induce the upregulation of differentiation-associated genes [25]. Second, keratinocytes were isolated from human epidermis and immediately subjected to single-cell RNA-sequencing (scRNA-seq) [29,30].

*HMOX1* was expressed only at minimal levels in the initial phase of keratinocyte differentiation in vitro and increased more than 100-fold on day 7 of the confluent culture (Figure 2B), when differentiation markers loricrin and filaggrin were also upregulated (Figure S1). scRNA-seq analysis of epidermal cells showed a high expression of loricrin, a marker of the epidermal granular layer, in terminally differentiated keratinocytes (Figure 2C). mRNA of *CYCS*, encoding the hemoprotein cytochrome c, was detected in all types of epidermal cells (Figure 2D). Of note, fully cornified, dead cells are not included in this analysis. *HMOX1* was detected at significant levels only in terminally differentiated keratinocytes (Figure 2E), indicating that its expression was induced in the late phase of differentiation in vivo. Of note, the shape of the violin plot with a maximal width around expression level 2 and a very narrow end at expression level 0 demonstrates that close to 100% of the loricrin-positive cells contain *HMOX1* mRNA. 

### 3.2. Immunolabeling Confirms Presence of HO-1 Protein in Terminally Differentiated Epidermal Keratinocytes 

**Figure 3:**
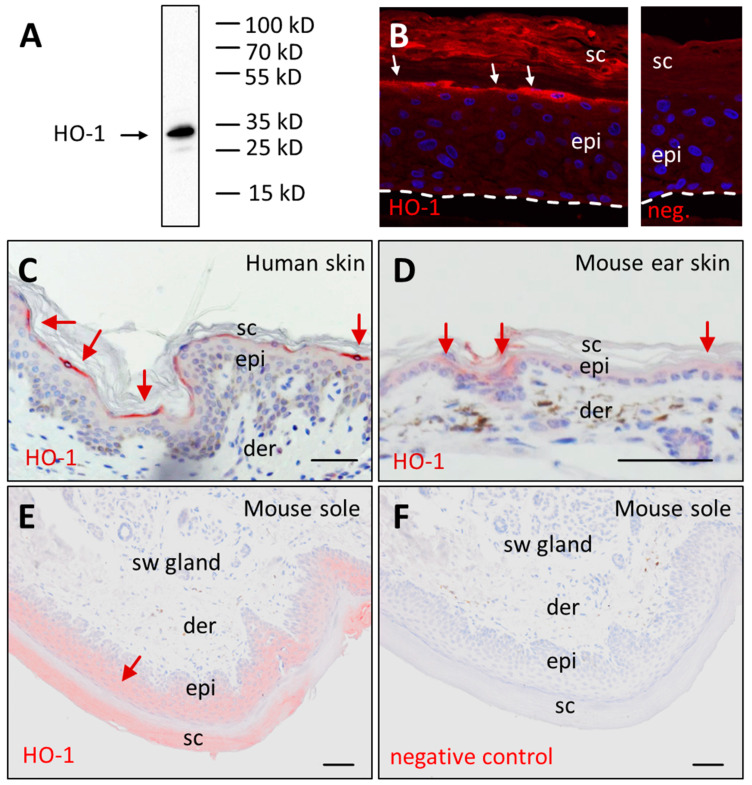
Heme oxygenase-1 (HO-1) protein is present in terminally differentiated keratinocytes. (**A**) The anti-HO-1 antibody was used in Western blot analysis of human keratinocytes differentiated in an in vitro skin equivalent. A band at 33 kilo-Daltons (kD), corresponding to the predicted molecular mass of HO-1, was detected. Positions of a molecular mass marker are indicated on the right. (**B**) Immunofluorescence labeling (red) of HO-1 in human skin equivalents. Immunohistochemical staining (red) of HO-1 in human skin (**C**), mouse ear skin (**D**) and mouse sole skin (**E**). (**F**) Negative control. Sections were counter-stained with the DNA stain Hoechst 33258 (blue) (**B**) and hematoxylin (blue) (**C**–**F**). Images are representative for *n* = 3 biological replicates. Scale bars, 50 µm. der, dermis; epi, epidermis; neg., negative control; sc, stratum corneum; sw, sweat.

To detect HO-1 protein, we used an antibody which specifically bound HO-1 extracted from the epidermal compartments of reconstructed human skin equivalents (Figure 3A; Figure S2) which contain keratinocytes differentiated in vitro [26]. Immunofluorescence analysis showed that HO-1 is present in the differentiated keratinocytes below the stratum corneum in reconstructed human skin equivalents (Figure 3B). Immunohistochemical staining of human abdominal skin revealed HO-1 expression in the outermost cells of the granular layer of the epidermis (Figure 3C). In the ear skin of the mouse, HO-1 was also detected, though with lower signal intensity, in suprabasal keratinocytes (Figure 3D). HO-1 was more evenly expressed in suprabasal epidermal keratinocytes of the mouse sole (Figure 3E), where the epidermis is considerably thicker than on the ear. HO-1 was detected neither in the mesenchymal cells of the dermis nor in the epithelial cells of the sweat glands (Figure 3E). Negative control experiments confirmed the specificity of the immunolabeling (Figure 3F). Thus, the investigation of HO-1 protein localization (Figure 3) is in agreement with the high abundance of *HMOX1* mRNA in differentiated keratinocytes (Figure 2), suggesting that transcriptional upregulation drives the accumulation of HO-1 in keratinocytes prior to cornification.

### 3.3. Development of Mouse Skin, Hair and Teeth Is Not Altered by Epithelial Deletion of HO-1

**Figure 4:**
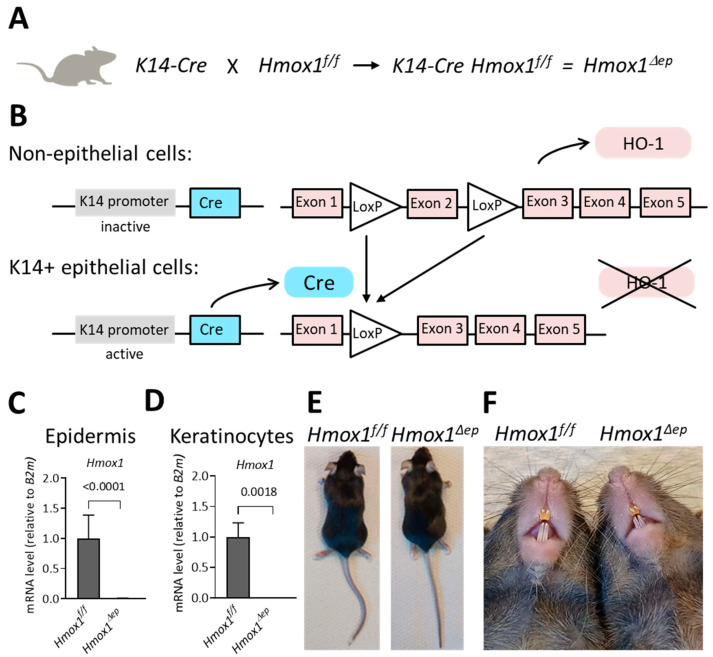
Epithelial deletion of heme oxygenase-1 (*Hmox1*). (**A**) *Hmox1^Δep^* mice were generated by crossing *K14-Cre* and *Hmox1^f/f^* mice. (**B**) Schematic depiction of Cre-mediated recombination in K14-positive epithelial cells, leading to the deletion of Hmox1 exon 2 and subsequent loss of HO-1 expression. RT-qPCR analysis of *Hmox1* mRNA levels in the epidermis (**C**) and isolated keratinocytes (**D**) of control (*Hmox1^f/f^*) and epithelial *Hmox1*-deleted (*Hmox1^Δep^*) mice. The mRNA levels are normalized to the mRNA levels of *B2m* in the same samples. *p*-values (*t*-test) are indicated above the horizontal bars. (**E**) Macroscopic appearance of *Hmox1^f/f^* and *Hmox1^Δep^* mice at an age of 3 months. Neither male (*n* > 10) nor female (*n* > 10) mice displayed phenotypic abnormalities. (**F**) Ventral view of *Hmox1^f/f^* and *Hmox1^Δep^* snout skin and teeth of mice at an age of 3 months. Note the absence of scaling or signs of skin inflammation. The yellow color of upper incisors indicates normal secretion of iron into the enamel.

To investigate the role of HO-1 in terminally differentiated keratinocytes, we crossed *Hmox1^f/f^* mice, in which exon 2 of *Hmox1* is flanked by *loxP* sites [22], with mice expressing Cre recombinase under the control of the keratin 14 (K14) promoter (*K14-Cre*). Expression of Cre in K14-positive epithelial cells leads to the deletion of *Hmox1* in stem cells of the epidermis and therefore also to the absence of *Hmox1* from all differentiated keratinocytes derived from these epithelial stem cells (*Hmox1^f/f^ K14-Cre*; *Hmox1^Δep^*) (Figure 4A,B). The K14-Cre-mediated recombination efficiently suppressed expression of *Hmox1* in the epidermis (Figure 4C) and in keratinocytes isolated from the epidermis of *Hmox1^Δep^* mice (Figure 4D). *Hmox1^Δep^* mice were born at the expected Mendelian ratios and did not develop overt macroscopic abnormalities during maintenance up to an age of 10 months (Figure 4E,F). The skin of *Hmox1^Δep^* mice showed neither aberrant scaling nor signs of inflammation. The iron-dependent yellow coloration of the upper incisors [31] was not impaired by the epithelial deletion of *Hmox1* (Figure 4F).

### 3.4. HO-1 Deletion Does Not Impair Terminal Differentiation under Standard Housing Conditions of Mice

**Figure 5:**
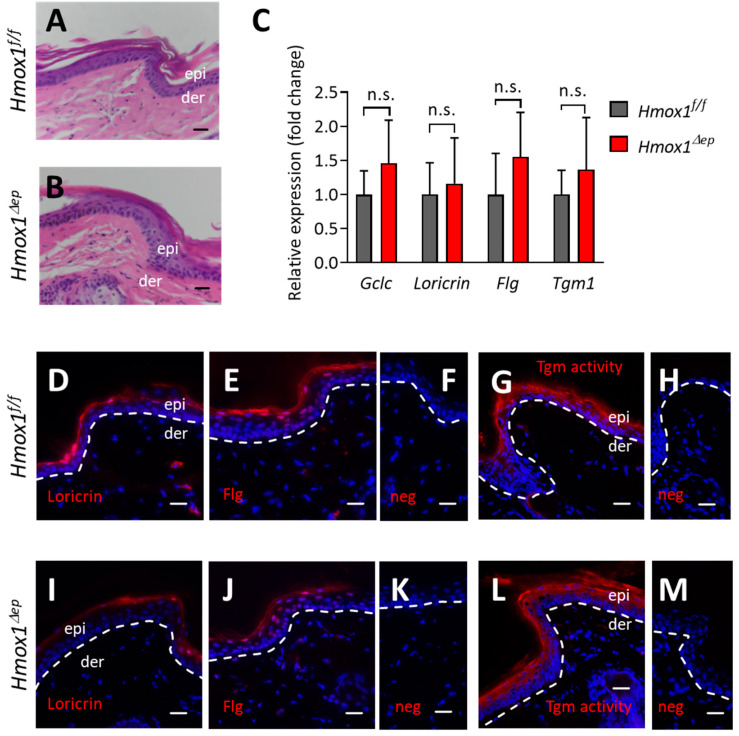
Epithelial deletion of heme oxygenase-1 (*Hmox1*) does not impair cornification. Sections through the tail skin of *Hmox1^f/f^* (**A**,**D**–**H**) and *Hmox1^Δep^* (**B**,**I**–**M**) mice at an age of 4–6 months were stained with hematoxylin and eosin (**A**,**B**), immunofluorescence-labelled (red) for loricrin (**D**,**I**), filaggrin (**E**,**J**) and labelled (red) for transglutaminase (Tgm) activity (**G**,**L**). In negative (neg) control experiments, the primary antibody was replaced by immunoglobulin from non-immunized animals (**F**,**K**) and calcium was replaced by EDTA (**H**,**M**). Sections were counter-stained with hematoxylin (blue) (**A**,**B**) and Hoechst 33258 (blue) (**D**–**M**). Images are representative for *n* = 3 mice per genotype. The dermo–epidermal junction is marked with a dashed-in line (**D**–**M**). Scale bars, 20 µm. (**C**) The mRNA levels of *glutamate-cysteine ligase catalytic subunit* (*Gclc*), *loricrin*, *filaggrin* (*Flg*) and *transglutaminase 1* (*Tgm1*) in the skin of *Hmox1^f/f^* (grey bars, *n* = 6) and *Hmox1^Δep^* (red bars, *n* = 7) mice were quantified by RT-PCR and normalized to *B2m*. n.s., not significant (*t*-test).

To characterize the skin of *Hmox1^Δep^* mice at the histological and molecular level, we performed hematoxylin and eosin (H&E) staining and investigated markers of keratinocyte differentiation. H&E staining showed that *Hmox1^Δep^* mice developed a morphologically normal stratum corneum (Figure 5A,B). RT-PCR revealed that the expression levels of loricrin, filaggrin and transglutaminase 1 were not significantly different in the skin of *Hmox1^Δep^* and control mice (Figure 5C). Likewise, expression of glutamate-cysteine ligase catalytic subunit (*Gclc*), a marker of the Nrf2-inducible response to oxidative stress, was not significantly altered in *Hmox1^Δep^* skin (Figure 5C), suggesting that a lack of anti-oxidative HO-1 did not cause oxidative stress. Consistent with the mRNA expression levels, filaggrin and loricrin proteins showed similar abundance in immunolabelling and their histological localization pattern was not altered in *Hmox1^Δep^* skin (Figure 5D–F,I–K). Finally, the distribution and intensity of transglutaminase activity was not altered by the absence of HO-1 (Figure 5G,H,L,M). These data suggest that, at least under the maintenance conditions for laboratory mice, apparently normal differentiation of epidermal keratinocytes is possible in the absence of HO-1.

## 4. Discussion

The establishment of the epidermal barrier function by the coordinated differentiation of keratinocytes during development and postnatal life is critical for the survival of mammals. Keratinocyte differentiation involves the upregulation of multiple genes, some of which are essential for normal barrier formation, such as serine protease inhibitor Kazal type 5 (SPINK5) [32] and ATP-binding cassette subfamily A (ABCA12) [33], whereas others, such as loricrin [34] and epiplakin [35], have redundant or dispensable roles in epidermal cornification under homeostatic conditions. Other differentiation-associated genes, such as gasdermin A (*GSDMA*), are not required for cornification [26,36], but have indispensable functions under particular forms of stress such as bacterial infection [36]. Of note, keratinocyte differentiation genes were primarily studied in humans and mice, revealing conserved gene functions but also inter-species differences [37]. Non-model species must be taken into consideration for conclusions about the epidermis of mammals in general [38,39,40,41]. In the present study, we showed that HO-1 is transcriptionally upregulated during terminal differentiation of epidermal keratinocytes, yet its expression in keratinocytes is dispensable for the development and postnatal function of the skin under standard housing conditions of mice. Our study was not designed to find conditions, possibly involving endogenous or exogenous disturbances of epidermal homeostasis or microbial dysbiosis, under which HO-1 might play an essential role. We can rather conclude that HO-1-mediated metabolism of heme, derived from hemoproteins or other sources, is not essential for homeostatic differentiation of keratinocytes in the mouse.

Our results demonstrate that HO-1 is constitutively expressed in human and murine epidermal keratinocytes prior to cornification, thereby confirming and extending the findings of previous reports [17,19]. The immunostaining of HO-1 was stronger in human epidermis than in mouse epidermis, which may be caused by higher expression levels in human keratinocytes or by higher binding affinity of the antibody against human HO-1. Using a beta-galactosidase reporter gene, the mouse *Hmox1* promoter was demonstrated to be consistently active in differentiated keratinocytes within stratified epithelia of murine skin, tongue and esophagus [19], indicating that the mouse is a useful model for the study of epithelial HO-1. In addition to our study of adult skin and cultured keratinocytes, we performed a preliminary investigation of embryonic skin development using gene expression data sets in the National Library of Medicine (NLM). We found that *Hmox1* is expressed in mouse skin on embryonic day E17.5, which is the time of initial cornification [42]. In mice lacking interferon regulatory factor 6 (Irf6), a transcription factor required for the development of differentiated epidermal layers and the periderm [43], *Hmox1* expression is reduced in parallel with the decreased expression of loricrin and filaggrin (Figure S3), suggesting that *Hmox1*, *Lor* and *Flg* are subjected to a similar, Irf6-dependent regulation in the developing epidermis. 

The late phase of keratinocyte differentiation involves a complete remodeling of the cell including the breakdown of mitochondria and other organelles [6,44,45]. It is conceivable that these processes affect the redox balance which is important for proper cornification [46]. The results of the present study suggest that HO-1 is either not involved in controlling oxidative stress during cornification or its function is redundant due to the presence of other anti-oxidant protection mechanisms such as HO-2 [46,47]. Importantly, epidermal HO-1 may have a critical function when the skin is exposed to high levels of oxidative stress [48,49,50,51,52]. Such conditions remain to be investigated in *Hmox1^Δep^* and control mice. Independent from the possible function of HO-1 in relation to oxidative stress, the HO-1-dependent release of iron from heme may be important for the epidermis. As iron was proposed to be recycled prior to cornification of keratinocytes [18], HO-1 may be responsible for one step of this recycling. *Hmox1^Δep^* mice will be a valuable experimental model for further studies of HO-1-dependent processes in the epidermis. 

## Data Availability

Data generated and analyzed in this study are included in this article.

## Supplementary Materials

The following supporting information can be downloaded at: https://www.mdpi.com/article/10.3390/jdb11010012/s1, Figure S1: Expression of filaggrin and loricrin in human keratinocytes cultured in vitro. Figure S2: Scan of the entire HO-1 Western blot membrane shown in Figure 3A. Figure S3: *Hmox1* is expressed during development of mouse skin on embryonic day E17.5 and its expression is reduced in parallel to that of loricrin and filaggrin upon deletion of interferon regulatory factor 6 (Irf6).
