# Supplementary material for: Heme Oxygenase-1 Is Upregulated during Differentiation of Keratinocytes but Its Expression Is Dispensable for Cornification of Murine Epidermis
Source: J Dev Biol. 2023 Mar 10;11(1):12. doi: 10.3390/jdb11010012 (PMC10058925; doi:10.3390/jdb11010012)
Supplement: Supplementary file 1 [file jdb-11-00012-s001.zip › jdb-2244819-supplementary.pdf]

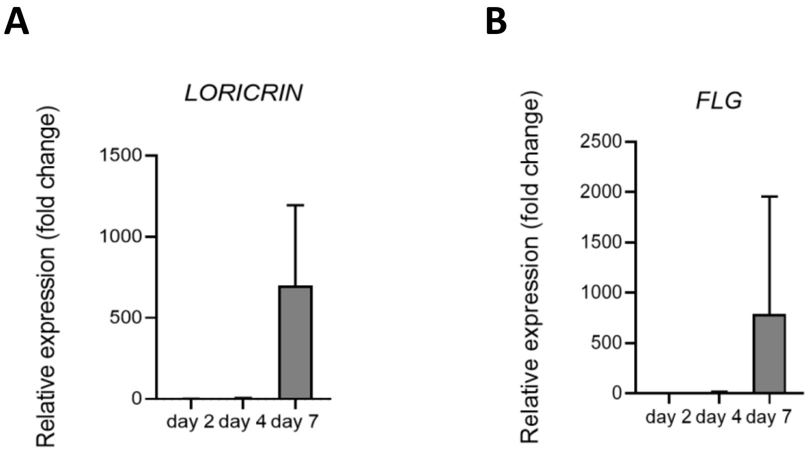

Figure S1. Expression of differentiation markers in human keratinocytes maintained in confluent culture. Primary human epidermal keratinocytes were cultured *in vitro*. RNA was prepared on days 2, 4 and 7 after cultures reached confluence, which enhances differentiation (Fischer et al. 2007). The RNA was subjected to RT-qPCR as described in the Materials and methods section. The graphs show the expression levels (fold change from day 2) of *LORICRIN* (**A**) and filaggrin (*FLG*) (**B**) relative to those of a house-keeping gene. The results of the *HMOX1* expression analysis of the same samples are shown in Figure 2B.

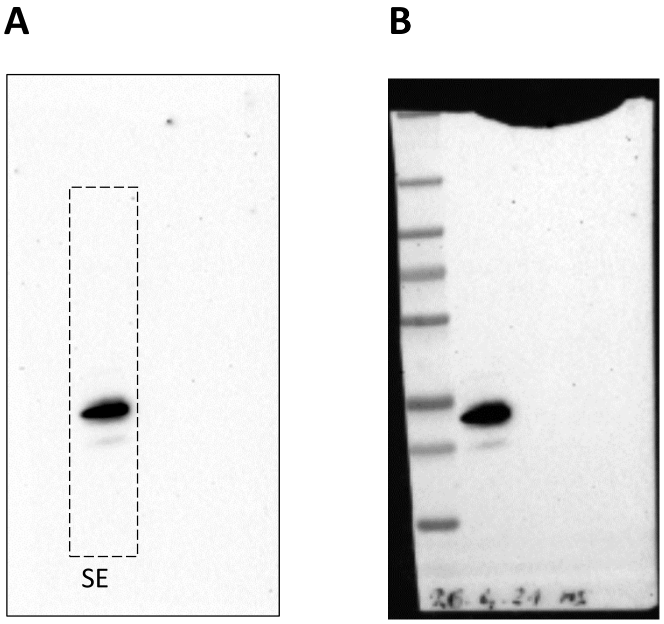

Figure S2. Scan of the entire HO-1 western blot membrane shown in Figure 3A. (**A**) Chemiluminesence signal of the entire membrane. (**B**) Chemiluminesence signal of the entire membrane merged with the light photography of the membrane to show the borders of the membrane and the pre-stained molecular weight marker. The rectangle (dashed line) in panel A marks the area shown in Figure 3A. This lane contains proteins from a human skin equivalent (SE). The purpose of the western blot was to test whether the anti-HO-1 antibody binds a protein of the expected mass. The band was detected at a molecular mass of 33 kD, confirming the specificity of the antibody.

Hmox1

Profile GDS2359 / 1448239_at
Title Interferon regulatory factor 6 null mutation effect on the skin
Organism Mus musculus

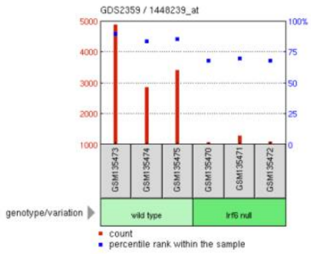

Graph caption help

Sample	Title	Value	Rank
GSM135473	IRF6 wt, biological rep1	4880.3	90
GSM135474	IRF6 wt, biological rep2	2861.3	84
GSM135475	IRF6 wt, biological rep3	3412.3	86
GSM135470	IRF6 knockout, biological rep1	1074.9	68
GSM135471	IRF6 knockout, biological rep2	1291.1	70
GSM135472	IRF6 knockout, biological rep3	1105.6	68

Lor

Profile GDS2359 / 1448745_s_at
Title Interferon regulatory factor 6 null mutation effect on the skin
Organism Mus musculus

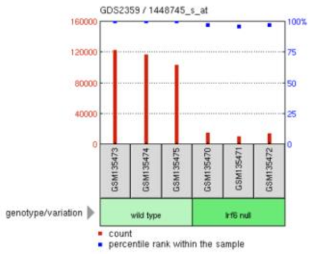

Graph caption help

Sample	Title	Value	Rank
GSM135473	IRF6 wt, biological rep1	123180	100
GSM135474	IRF6 wt, biological rep2	117545	100
GSM135475	IRF6 wt, biological rep3	103568	100
GSM135470	IRF6 knockout, biological rep1	16358.1	97
GSM135471	IRF6 knockout, biological rep2	10809.1	96
GSM135472	IRF6 knockout, biological rep3	14699.8	97

Flg

Profile GDS2359 / 1427268_at
Title Interferon regulatory factor 6 null mutation effect on the skin
Organism Mus musculus

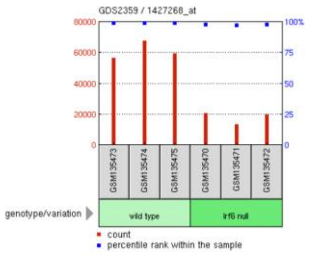

Graph caption help

Sample	Title	Value	Rank
GSM135473	IRF6 wt, biological rep1	56730.7	99
GSM135474	IRF6 wt, biological rep2	67561.2	99
GSM135475	IRF6 wt, biological rep3	59744.6	99
GSM135470	IRF6 knockout, biological rep1	21149.5	98
GSM135471	IRF6 knockout, biological rep2	13726.9	97
GSM135472	IRF6 knockout, biological rep3	20012.7	98

Krt14

Profile GDS2359 / 1423935_x_at
Title Interferon regulatory factor 6 null mutation effect on the skin
Organism Mus musculus

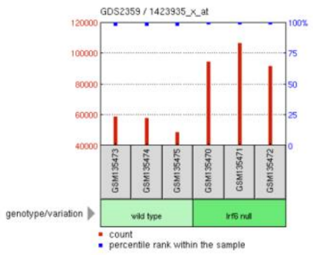

Graph caption help

Sample	Title	Value	Rank
GSM135473	IRF6 wt, biological rep1	58808.3	99
GSM135474	IRF6 wt, biological rep2	58011.8	99
GSM135475	IRF6 wt, biological rep3	48896.9	99
GSM135470	IRF6 knockout, biological rep1	94719	100
GSM135471	IRF6 knockout, biological rep2	106902	100
GSM135472	IRF6 knockout, biological rep3	91688.8	100

Krt10

Profile GDS2359 / 1452166_a_at
Title Interferon regulatory factor 6 null mutation effect on the skin
Organism Mus musculus

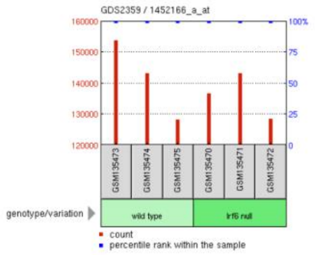

Graph caption help

Sample	Title	Value	Rank
GSM135473	IRF6 wt, biological rep1	153870	100
GSM135474	IRF6 wt, biological rep2	143243	100
GSM135475	IRF6 wt, biological rep3	128401	100
GSM135470	IRF6 knockout, biological rep1	136808	100
GSM135471	IRF6 knockout, biological rep2	143219	100
GSM135472	IRF6 knockout, biological rep3	128522	100

Krt16

Profile GDS2359 / 1448932_at
Title Interferon regulatory factor 6 null mutation effect on the skin
Organism Mus musculus

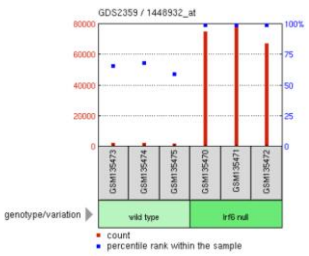

Graph caption help

Sample	Title	Value	Rank
GSM135473	IRF6 wt, biological rep1	997.3	66
GSM135474	IRF6 wt, biological rep2	1129.7	68
GSM135475	IRF6 wt, biological rep3	694	59
GSM135470	IRF6 knockout, biological rep1	74846.9	99
GSM135471	IRF6 knockout, biological rep2	78088.6	99
GSM135472	IRF6 knockout, biological rep3	66991.9	99

Figure S3. *Hmox1* is expressed during development of mouse skin on embryonic day E17.5 and its expression is reduced in parallel to that of *loricrin* and *filaggrin* upon deletion of interferon regulatory factor 6 (Irf6). Gene expression omnibus (GEO) dataset GDS2359, comprising microarray-based gene expression analysis of wildtype and *Irf6*-deficient mouse skin at embryonic day E17.5, was analyzed for the expression levels of *Hmox1*, two markers of keratinocyte terminal differentiation (loricrin, *Lor*; filaggrin, *Flg*), a basal layer keratin (*Krt14*), a suprabasal keratin (*Krt10*) and a stress-associated keratin (*Krt16*). *Irf6*-null mice lack terminally differentiated epidermal layers and the periderm (Ingraham et al. 2006). Note that expression of *Lor*, *Flg* and *Hmox1* is similarly reduced by *Irf6* deletion. The National Library of Medicine (NLM) is acknowledged for providing GEO profiles in the public domain for free distribution. Reference: Ingraham CR, Kinoshita A, Kondo S, Yang B, Sajan S, Trout KJ, Malik MI, Dunnwald M, Goudy SL, Lovett M, Murray JC, Schutte BC. Abnormal skin, limb and craniofacial morphogenesis in mice deficient for interferon regulatory factor 6 (Irf6). *Nat Genet.* **2006**, *38*, 1335-1340. doi: 10.1038/ng1903.
